# Analysis of Proteome Dynamics in Early-Stage Sporulation-Inhibited Variants of *Parageobacillus thermoglucosidasius*

**DOI:** 10.3390/ijms262311735

**Published:** 2025-12-04

**Authors:** Marie Millgaard, Oihane Irazoki, Viviënne Mol, Ivan Pogrebnyakov, Alex Toftgaard Nielsen

**Affiliations:** The Novo Nordisk Foundation Center for Biosustainability, Technical University of Denmark, 2800 Kongens Lyngby, Denmark; marmill@dtu.dk (M.M.); oihira@biosustain.dtu.dk (O.I.); vim@again.bio (V.M.); ivpro@biosustain.dtu.dk (I.P.)

**Keywords:** proteomics, sporulation, thermophile, *Parageobacillus thermoglucosidasius*

## Abstract

*Parageobacillus thermoglucosidasius* is a thermophilic Gram-positive that is emerging as a platform for bioproduction. However, one potential limitation of *P. thermoglucosidasius* is its natural tendency to sporulate. As a non-model organism, the complex regulatory system that governs sporulation in *P. thermoglucosidasius* remains poorly characterised. To advance current understanding, this study presents a comparative characterisation and proteomic analysis of the *P. thermoglucosidasius* wildtype strain alongside four early-stage sporulation-inhibited variants. To inhibit sporulation, the genes *spo0A*, *spo0B*, *spo0F*, and *sigF* were targeted for deletion, as based on their crucial regulatory roles in the sporulation pathway of *Bacillus subtilis*. Microscopic analysis indicated that the Δ*spo0A*, Δ*spo0F*, and Δ*sigF* strains were sporulation-suppressed while the Δ*spo0B* strain sporulated at low levels. Proteomics data were obtained from four different growth stages and the resulting expression profiles were compared. Consistent with the regulatory network of *B. subtilis*, the Δ*spo0A*, Δ*spo0B*, and Δ*spo0F* strains exhibited largely inactive sporulation pathways, while the Δ*sigF* strain retained some early regulatory functions. Additionally, two co-expression modules comprising approximately 300 genes were identified and linked to the *P. thermoglucosidasius* sporulation pathway. Overall, these results expand the understanding of the sporulation network of *P. thermoglucosidasius* and provide a foundation for future engineering of non-sporulating variants.

## 1. Introduction

Fossil-based production strategies currently dominate the chemical industry, leading to concerns regarding the environmental damage caused by the resulting greenhouse gas emissions. As population numbers and living standards continue to rise, the scope and scale of the chemical sector steadily grows [1]. As such, there is an increasing demand to transition towards more sustainable industrial production practices. Fermentation-based approaches have been proposed as a compelling alternative, as they rely on renewable feedstocks instead of fossil-based ones. However, for biochemicals to endure on the global market, their production must be competitive with conventional petrochemical strategies [1,2].

To advance biochemical fermentation technology, one approach explores the potential of thermophilic microorganisms as production hosts. There are many benefits associated with high-temperature fermentation, including decreased cooling cost, reduced contamination risks, higher reaction rates, and improved purification of volatile products through evaporation [3,4]. Amongst thermophiles, *Parageobacillus thermoglucosidasius* has gained traction as a host for chemical production. *P. thermoglucosidasius* is a Gram-positive species of bacteria with an optimal growth temperature of around 60 °C [3,5]. In contrast to many thermophiles, *P. thermoglucosidasius* is responsive to genetic engineering strategies, which has allowed for the development of *P. thermoglucosidasius* strains for the production of a variety of compounds, such as ethanol [6,7,8], 2,3-butanediol [9,10], and 1-butanol [11]. However, despite its growing reputation, the status of *P. thermoglucosidasius* as a non-model organism inevitably means that there are many aspects of its metabolic and regulatory networks that currently remain elusive.

As an endospore-forming species, one major regulatory system of *P. thermoglucosidasius* relates to the process of sporulation. Sporulation is a survival strategy that naturally occurs in a broad range of bacterial species [12]. It usually arises as a stress response to adverse conditions, with the most common factor being nutrient starvation [13,14]. Mature endospores are metabolically dormant but highly resistant towards extreme environmental stresses. This allows the endospores to persist in harsh environmental conditions until a favourable change occurs, after which the spores may germinate and return to the vegetative cycle [13,15].

While useful for survival in nature, sporulation is generally considered to be a challenge under industrial settings. The regulatory process directs the host cell to expend valuable resources on endospore development, rather than growth or production. The high resistance of endospores also makes them prone to cause contaminations, as they may survive conventional sterilisation approaches. For these reasons, it is generally not desired for a production host to maintain an active sporulation pathway. It has recently been discovered that the deletion of sporulation regulator Spo0A suppresses the sporulating phenotype in *P. thermoglucosidasius* [16]. This indicates that the sporulation initiation system in *P. thermoglucosidasius* resembles that of its more well-characterised relative *Bacillus subtilis*. In *B. subtilis*, the deletion of the *spo0A* gene has previously been demonstrated to suppress sporulation [17]. However, Spo0A is also predicted to be a key regulator that exerts direct control over 121 genes and indirectly influences the expression of hundreds more [18]. If sporulation initiation in *P. thermoglucosidasius* is truly similar, it is necessary to consider that *spo0A* deletion is likely to cause a major shift in its regulatory network. Therefore, it is important to understand the impact of these kinds of regulatory deletions when developing a sporulation-suppressed variant of *P. thermoglucosidasius* for production purposes.

**Figure 1:**
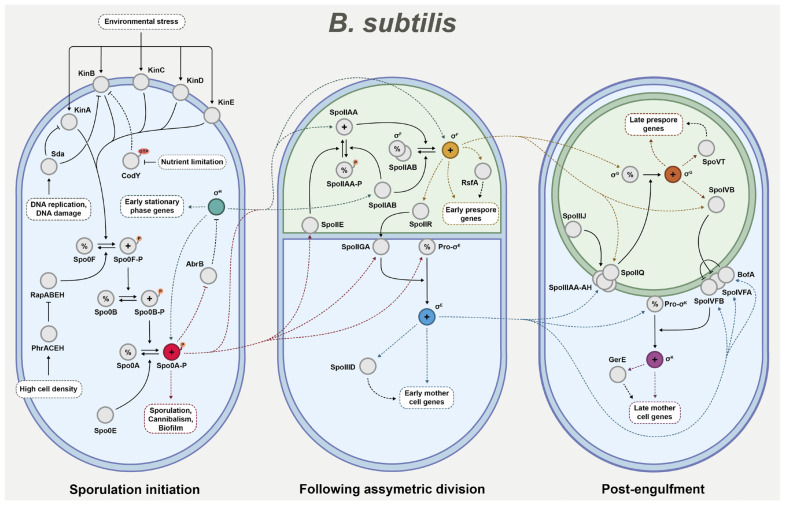
A simplified overview of the key regulatory mechanisms that initiate and progress sporulation in *B. subtilis*, as split into three phases based on the sequential activation of the sigma factor cascade. (+) indicates activated proteins, while (%) indicates inactivated ones. Dashed lines indicate transcriptional regulation, while solid lines indicate post-translational interactions.

When investigating the impact of sporulation regulator deletions, it is helpful to have a fundamental understanding of the broader context that these regulators operate within. While the sporulation pathway of *P. thermoglucosidasius* has yet to be characterised, the corresponding pathway of *B. subtilis* is better understood [13,19,20]. As such, one can study the *B. subtilis* network [19,20] to gain more insight into the potential functionality of the *P. thermoglucosidasius* counterpart. To provide an overview of sporulation regulation in *B. subtilis*, the key regulatory steps can roughly be divided into three phases based on the precise sequential activation of a cascade of sigma factors [14,21,22]. The first phase controls the initiation of the sporulation pathway, the second phase establishes compartmentalised gene expression following the asymmetric division of the cell into mother cell and prespore, and the third phase guides the final stages of endospore maturation following engulfment (Figure 1).

**Phase 1—Sporulation initiation**: In the vegetative cell, the decision to enter the sporulation pathway is chiefly governed by the activation of key transcription factor Spo0A through phosphorylation [14]. Besides its central role in sporulation, Spo0A activation has been linked to several other processes, most notably biofilm formation and cannibalism. The concentration of phosphorylated Spo0A present in the cell determines which of these developmental programmes the cell may undertake [13,23,24]. In the case of sporulation, high levels of phosphorylated Spo0A is required for initiation to occur [13,15].

Spo0A phosphorylation is controlled through a complex phosphorelay signal transduction system. Central to this system are the phosphotransferases Spo0F and Spo0B, which act as intermediates for Spo0A phosphorylation. The Spo0F protein phosphorylates Spo0B, which in turn phosphorylates Spo0A [14,15,24]. The phosphorelay is primarily governed by five histidine kinases KinABCDE, of which KinA and, to a lesser extent, KinB are considered most involved in sporulation initiation [15,25]. These kinases autophosphorylate in response to various environmental stresses that currently remain unidentified [13,15]. Once phosphorylated, the kinases facilitate the phosphorylation of Spo0F, and thus the system is pushed towards sporulation induction. Several factors may intercept the kinases and thereby delay the onset of sporulation. One notable example is Sda, which blocks KinA, and potentially KinB, in response to DNA damage or replication stress [15]. Another example is CodY, which binds GTP and branched-chain amino acids to repress *kinB* and other early sporulation-associated genes. Once nutrient deprivation occurs, CodY can no longer enact the repression and sporulation is consequently promoted [15,24,26]. There are also negative regulators that directly affect the core proteins of the phosphorelay. Examples include phosphatases Spo0E and RapABEH which target Spo0A and Spo0F, respectively. RapABEH are in turn inhibited by imported Phr pentapeptides that serve as indicators of high cell density [14,15,24].

Once the Spo0A phosphorylation level passes the threshold for sporulation activation, it induces a major shift in the cell regulatory network, which includes the positive regulation of several genes crucial for advancing the sporulation pathway [18,25]. Another key regulator during this early stage is the sigma factor σ^H^. Through interactions with RNA polymerases, this sigma factor regulates the transcription of early stationary phase genes, including some associated with sporulation [25,27]. The regulatory functions of Spo0A and σ^H^ are closely interlinked, and both are known to positively regulate each other. Namely, *spo0A* is upregulated as part of the σ^H^ regulon [27], and in turn, Spo0A inhibits the AbrB transcriptional regulator that would otherwise repress the σ^H^ gene [15,28]. Eventually, the wide array of regulatory changes will facilitate axial filamentation, resulting in the asymmetric division of the cell into prespore and mother cell [13,25,29]. This sets the stage for the next regulatory development in the sporulation programme.

**Phase 2—After asymmetric division**: Upon completion of asymmetric division, different gene expression programmes are established in the two resulting cells. This compartmentalisation of gene expression is driven by the cell-specific sigma factors σ^F^ and σ^E^, which are active in the prespore and mother cell, respectively. Interestingly, both are present in the cell prior to asymmetric division, but are kept inactive until the formation of the septum [13,24,29].

σ^F^ is synthesised under the control of both Spo0A and σ^H^, and is the primary regulator in the prespore [18,27]. Prior to division, σ^F^ is bound and held inactive by anti-sigma factor SpoIIAB. Once the septum forms, phosphatase SpoIIE activates anti–anti-sigma factor SpoIIAA, which then releases σ^F^ by binding SpoIIAB [13,24]. It is notable that SpoIIAB is also able to deactivate SpoIIAA through phosphorylation, thus providing a more indirect strategy for σ^F^ inhibition [25]. Through this regulatory system, σ^F^ is activated exclusively in the prespore, though the exact mechanism behind this prespore-specific activation is still under study [15,29]. Once σ^F^ is activated, it initiates the transcription of around 50 genes in the early prespore, including transcriptional regulator RsfA which co-controls the regulation of various σ^F^-dependent genes [29]. Importantly, the *spoIIR* gene is activated, which serves an instrumental role in the activation of key sigma factor σ^E^ in the mother cell [25,30].

σ^E^ is originally synthesised as an inactive precursor under the transcriptional control of Spo0A [18]. The precursor is activated through interactions with the membrane-bound protease SpoIIGA in the mother cell [29]. However, to facilitate this reaction, the σ^E^-regulated SpoIIR protein must activate the SpoIIGA across the septum [13,24,29]. The necessity of this intercompartmental signalling step exemplifies the fact that the developmental programmes occurring in the two cells are not in isolation [24,25]. Communication between the prespore and the mother cell is instead a crucial part of endospore formation. Once active, σ^E^ facilitates the transcription of over 200 genes to progress the developmental programme of the mother cell, including initiation of engulfment and spore coat assembly [25,30]. Transcriptional regulator SpoIIID is also activated in order to support the regulation of certain σ^E^-dependent genes [21,25,29].

**Phase 3—Post-engulfment**: Upon the completion of engulfment, the prespore exists as a protoplast contained within the mother cell cytosol [13,24]. At this point, the final two sigma factors σ^G^ and σ^K^ are activated to direct the final steps of endospore formation.

Prior to engulfment, σ^G^ is synthesised in the prespore under the transcriptional control of σ^F^, but is held in an inactive state through unknown means [13,24,30]. Activation of σ^G^ relies on the σ^F^-regulated protein SpoIIQ and a complex consisting of eight σ^E^-regulated proteins SpoIIIAA-AH [13]. It is suggested that these proteins together form a ‘feeding tube’ channel that enables the transfer of molecules between the mother cell and the prespore, and that the activation of σ^G^ is dependent on this transfer in some way [13,15,31]. The SpoIIIJ translocase has been found to be essential for σ^G^ activation as well, potentially due to being involved with the membrane insertion of the channel [25,29,31]. Once activated, σ^G^ initiates the transcription of genes that facilitate late-stage processes, including DNA damage protection and germination preparations [25]. It also includes the initiation of transcriptional regulator SpoVT to help control σ^G^-dependent regulation [25,29].

σ^K^ is the final sigma factor in the cascade, as found in the mother cell under σ^E^ transcriptional control. σ^K^ is synthesised as an inactive precursor that needs to be processed by the metalloprotease SpoIVFB in order to activate [13,24]. However, SpoIVFB is initially inhibited by BofA, as facilitated by SpoIVFA linking the two proteins together in a membrane-bound complex. σ^K^ activation is triggered by σ^G^-regulated protease SpoIVB, which relieves SpoIVFB inhibition by cleaving the SpoIVFA proteins [13,15,29]. When finally activated, σ^K^ directs the transcription of genes related to the late-stage developments of sporulation, such as spore coat assembly and spore maturation [25]. It also activates the transcriptional regulator GerE to support σ^G^-regulation during the final steps of endospore formation [25,29].

### Targeting Sporulation in P. thermoglucosidasius

Similarly to *B. subtilis*, sporulation in *P. thermoglucosidasius* is a complex regulatory process that oversees major changes in expression and morphology over the course of endospore formation. In *B. subtilis*, sporulation is also known to be a committed process [15,32], meaning that the pathway becomes irreversible at a certain stage. This point of commitment is thought to occur around the activation of σ^E^ in the mother cell. To avoid trapping the cells in a state of arrested development, it would therefore be preferable to target regulatory genes that are employed prior to this step.

While little experimental characterisation of the *P. thermoglucosidasius* metabolic network currently exists, several databases offer genome annotations based on comparative methods, such as sequence homology. Through database annotations, predicted homologues to most of the primary *B. subtilis* regulators seen in Figure 1 have been identified in *P. thermoglucosidasius*, including all sigma factors involved in the cascade. Working from the assumption that the cascade operates as in *B. subtilis*, this study aims to investigate the impact of deleting pre-commitment regulators Spo0A, Spo0B, Spo0F, and σ^F^ in *P. thermoglucosidasius*. Here, we assess the influence of each deletion on growth and sporulation, and we perform a proteomic analysis to identify sporulation-related genes in *P. thermoglucosidasius*. This study aims to advance our understanding of the sporulation pathway in Gram-positive thermophilic bacteria, while contributing to future strain development by providing a characterization of the regulatory network in *P. thermoglucosidasius* sporulation-inhibited variants.

## 2. Results

### 2.1. Growth and Microscopy Analysis

**Figure 2:**
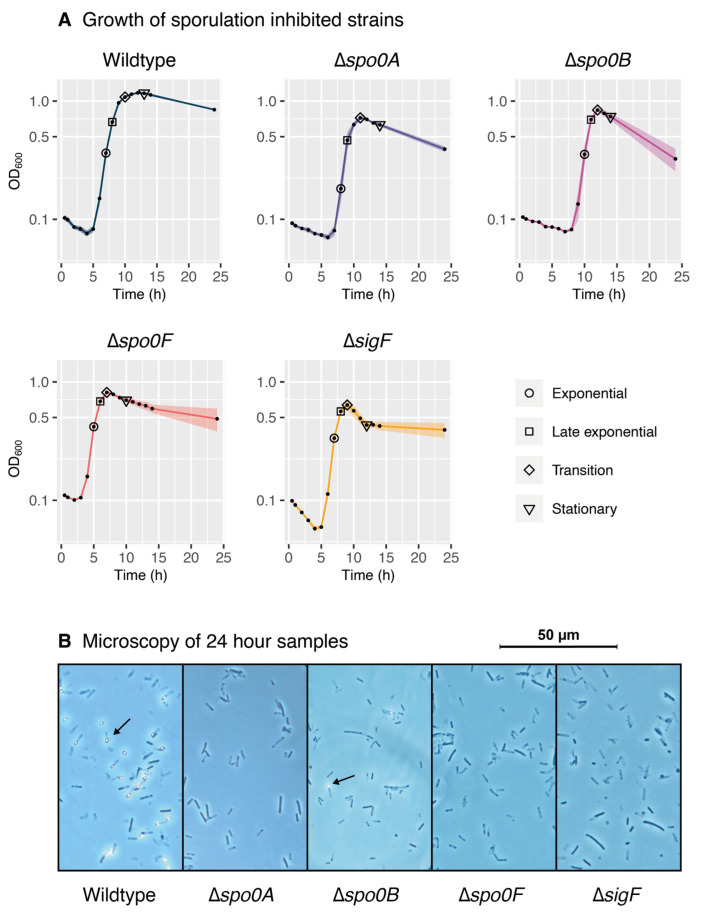
Characteristics of wildtype and sporulation-inhibited strains of *P. thermoglucosidasius* following growth on TMM supplied with 1 g/L yeast extract. (**A**) Growth of the five strains over the course of 24 h of culturing. During the experiment, proteomic samples were collected from each culture to represent four different growth phases, as indicated by the legend. (**B**) Microscopy images of wildtype and sporulation-inhibited strains following 24 h of culturing in TMM with 1 g/L yeast extract. Examples of sporulation are indicated by arrows. High levels of spores were observed in the wildtype cultures, while low levels of spores were observed in the Δ*spo0B* strain. No signs of spores were observed with the other strains.

In this study, we constructed four early-stage sporulation-inhibited variants of *P. thermoglucosidasius* through the deletion of genes homologous to the *B. subtilis* key sporulation regulators Spo0A, Spo0B, Spo0F, and σ^F^. To test for differences in growth patterns between the wildtype and sporulation-inhibited strains, each strain was inoculated in Thermophile Minimal Medium (TMM) with yeast extract and grown over a period of 24 h. Following inoculation, all strains displayed a notable lag phase as the cells adapted to the new medium (Figure 2A). The length of the lag-phase varied between the strains, with the shortest being the Δ*spo0F* strain at around 3 h, and the longest being the Δ*spo0B* strain at around 7 h. As the deletion of sporulation regulators is expected to predominantly affect the later growth phases, it is difficult to assess how much impact these mutations had on the lag phase and how much can be attributed to natural variance. In addition, all cultures also displayed an early reduction in OD600 during the lag phase. This pattern is likely due to the initial shift from a rich pre-culture medium to a less abundant culturing medium. During the subsequent lag phase, some cells may lose viability while performing the necessary physiological adjustments to adapt to the new medium. Notably, the Δ*sigF* strain experiences a significantly steeper reduction in OD600 compared to the other strains, indicating that these cells may not cope well with adaptive stress.

Due to the connection between nutrient starvation and sporulation, it is to be expected that the major differences would be observed around the transition and stationary phases. In the case of the wildtype strain, it peaks at an OD600 of around 1.2 with a steady transition from growth to the stationary phase. The sporulation-inhibited strains failed to reach similar OD600 values, with the Δ*spo0B* strain reaching the highest peak at around OD 0.85, and the Δ*sigF* strain reaching the lowest peak at around OD 0.6. In contrast to the gradual transition and decline of the wildtype strain, the sporulation-inhibited strains exhibit a steeper decline in cell density following the peak, with the most severe instance being the Δ*sigF* strain once again. After 24 h of incubation, the wildtype retained an endpoint OD600 of approximately 0.85, while the sporulation-inhibited strains generally settled below OD 0.5.

To investigate the sporulation capacity of each strain, samples were collected after 24 h of growth and examined through phase contrast microscopy (Figure 2B). Predictably, the wildtype cultures displayed a high abundance of spores, while no spores were observed in the Δ*spo0A*, Δ*spo0F*, and Δ*sigF* cultures. Interestingly, most cells in the Δ*spo0B* cultures appeared to be non-sporulating, but each culture still managed to produce a small number of cells exhibiting a sporulating phenotype. This suggests that the Δ*spo0B* strain has an alternative sporulation initiation strategy that bypasses Spo0B in the phosphorelay, though it appears to be less effective than the conventional strategy based on the relatively low number of cells observed to be sporulating.

To further study these different strain behaviours, proteomic samples were collected at various time points to represent the exponential, late exponential, transition, and stationary growth phases of each strain. The sampling points are indicated in Figure 2A and listed in Table S7.

### 2.2. Principal Component Analysis (PCA)

**Figure 3:**
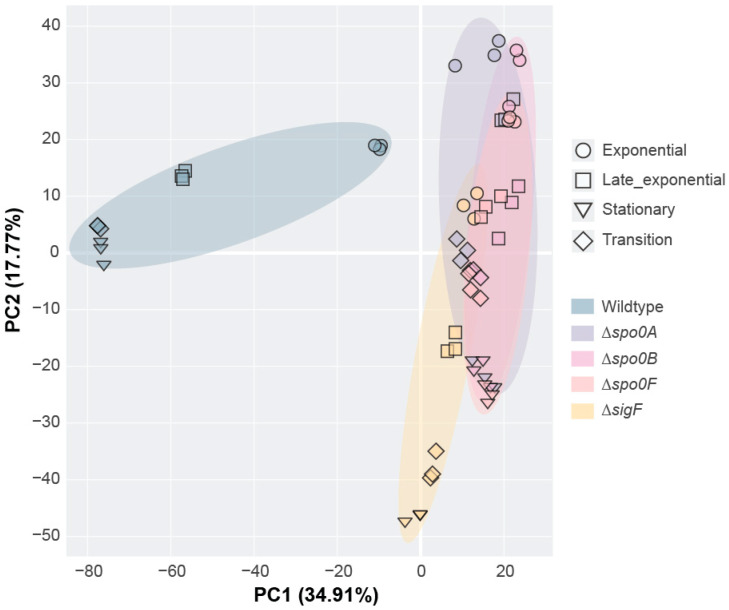
Principal Component Analysis (PCA) plot representing the variability in proteomic expression profiles across the five *P. thermoglucosidasius* strains and growth phases.

We applied PCA to the proteomics data from samples collected during the growth experiment (Figure 2A) to observe differences and correlations in protein expression between strains and growth phases. A PCA plot from this analysis is shown in Figure 3. As expected, we observed clear differences in protein expression between the wildtype and sporulation-inhibited strains. While the sporulation-inhibited strains generally cluster together, the wildtype remains entirely distinct, with the progression through each growth phase only increasing the separation. This speaks to the impact that sporulation leaves on the global proteomic profile of *P. thermoglucosidasius*, particularly in the later stages of growth. In contrast, the Δ*spo0A*, Δ*spo0B*, and Δ*spo0F* strains appear to be closely associated across all growth phases. This suggests that these strains share highly similar proteomic profiles, which is consistent with the tightly interlinked functionality of their respective regulators. Interestingly, the Δ*sigF* strain produces a somewhat different expression profile to that of the other sporulation-inhibited strains. While still close compared to that of the wildtype, this separation is likely a reflection of the differences in regulatory roles between σ^F^ and the three key regulators of the phosphorelay.

### 2.3. Sporulation Sigma Factor Cascade Characterisation

**Figure 4:**
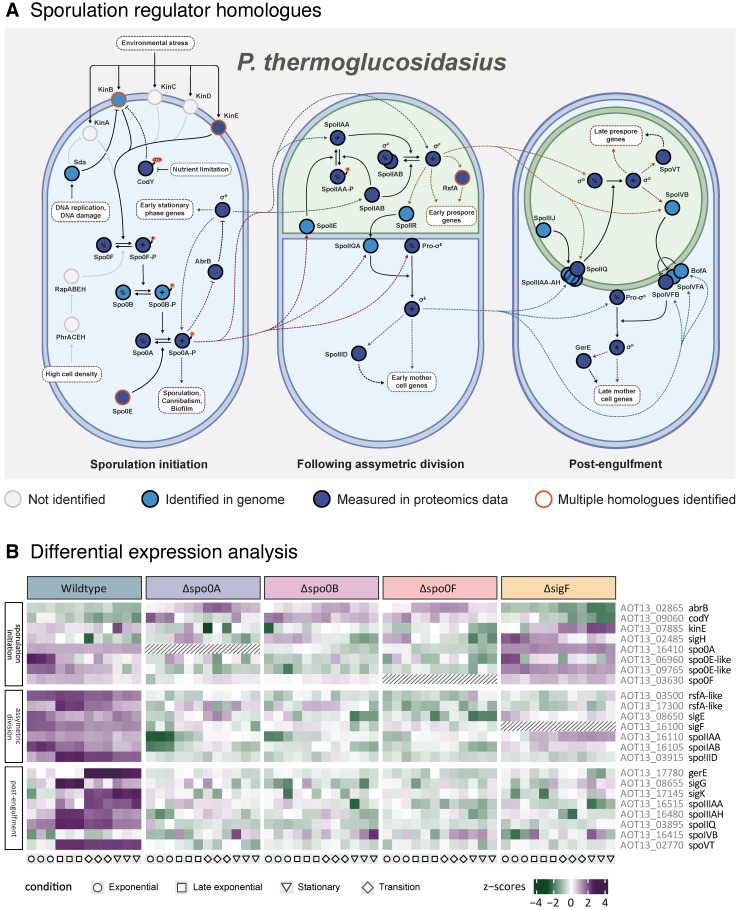
Analysis of sporulation regulator homologues identified in *P. thermoglucosidasius*. (**A**) An overview of the different putative sporulation regulators found to be present in *P. thermoglucosidasius*. While most of these key regulators have one or more identified homologues in the *P. thermoglucosidasius* genome, not all were measured in the proteomics data collected during this study. Dashed lines indicate transcriptional regulation, while solid lines indicate post-translational interactions. (**B**) Relative expression levels of the identified sporulation regulators in *P. thermoglucosidasius* across wildtype and sporulation-inhibited strains during different stages of growth. Purple denotes higher-than-average expression within the module (positive Z-scores), while green denotes lower-than-average expression (negative Z-scores).

As previously mentioned, several proteins have been identified as homologues to key *B. subtilis* sporulation regulators in *P. thermoglucosidasius*. To investigate the expression profiles of the sporulation regulator homologues that were identified in the proteomics data (Figure 1 and Figure 4A), a differential expression analysis was performed. The relative expression levels across strains and growth phases for the identified homologues have been visualised in Figure 4B. The Spo0B protein could not be quantified; however, while the expression profile of Spo0B therefore remains unknown, it can be presumed to resemble that of Spo0A and Spo0F, based on their closely linked regulatory roles.

When observing the expression profiles of the different phases in the wildtype strain, a pattern that broadly resembles the *B. subtilis* sporulation regulation network is visible. To start, it appears that sporulation had been initiated in the cultures by the time the earliest samples were collected during mid-exponential growth. Continuous upregulation of Spo0F and Spo0A, as well as downregulation of the sporulation inhibitors AbrB and CodY indicate an active phosphorelay. In addition, both σ^F^ and σ^K^ exhibit high levels of relative expression in the exponential samples, suggesting that asymmetric division had already occurred. This is further emphasised by the increased expression displayed by the other regulators associated with asymmetric division. Transcription of σ^G^ appears to have been initiated by the late exponential phase, alongside post-engulfment regulators SpoVT, SpoIIQ, and SpoIIIAH. At the transition phase, the final sigma factor σ^K^ was observed to be highly upregulated and active, as reflected by the concurrent upregulation of co-regulator GerE. Interestingly, the expression of σ^G^ appears to be downregulated at this stage, for reasons currently unknown. It is, however, possible that the extraction of certain prespore-specific proteins becomes more cumbersome as endospore maturation progresses.

When comparing the relative expression profiles of the sporulation-inhibited strains and the wildtype, it becomes apparent that the Δ*spo0A*, Δ*spo0B*, and Δ*spo0F* strains were highly similar. Notably, the proteins that upregulate in the wildtype generally appear to remain downregulated in these three strains, demonstrating the inactivation of the sequential cascade. Conversely, the early regulators AbrB and CodY were consistently upregulated in the Δ*spo0A*, Δ*spo0B*, and Δ*spo0F* strains, but not in the wildtype, reflecting their roles as sporulation repressors. Interestingly, the Δ*sigF* strain differs from the other sporulation-inhibited variants, as its early-phase regulators instead display expression profiles highly similar to those of the wildtype. This is most pronounced in the sporulation initiation phase, which aligns well with what is known about σ^F^ in *B. subtilis* [13]. Since both σ^F^ transcription and functionality are directly tied to Spo0A activation and occur downstream of the sporulation initiation network, the Δ*sigF* strain is the only sporulation-inhibited variant that retains a non-disrupted Spo0A phosphorelay. This is reflected in the close resemblance between the sporulation initiation profiles of the wildtype and Δ*sigF* strain, as well as in the apparent upregulation of SpoIIAA and Spo0IIAB, which in *B. subtilis* are known to be co-expressed with the *sigF* gene under the control of Spo0A and σ^H^. However, proteins downstream of σ^F^ generally fail to upregulate in the Δ*sigF* strain, resulting in a post-engulfment profile that resembles the other sporulation-inhibited strains more than the wildtype.

Based on the information about sporulation regulation and growth phases of the analysed strains, it seems likely that the *B. subtilis* pathway is a suitable model for endospore formation in *P. thermoglucosidasius*, at least in the context of the core regulatory network.

### 2.4. Weighted Gene Co-Expression Network Analysis (WGCNA)

**Figure 5:**
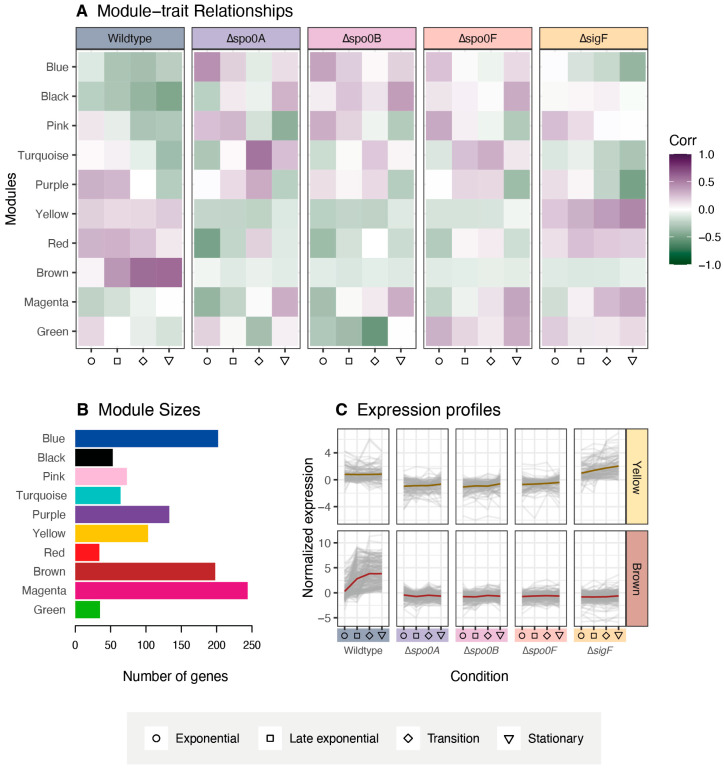
WGCNA of protein expression in wildtype and sporulation-inhibited *P. thermoglucosidasius* strains across four different growth phases. (**A**) Module-trait associations based on Pearson correlation coefficients. The colour indicates the correlation between the modules and traits. (**B**) Number of proteins assigned to each module. (**C**) Protein expression profiles of the Brown and Yellow modules.

WGCNA enables the identification of modules of highly co-expressed genes, while also providing information about the relationships between these modules and external traits, such as sample phenotypes. Here, we applied the method to identify proteins with similar expression patterns that are directly or indirectly affected by the sporulation-inhibiting gene deletions. The resulting analysis produced ten modules (named after colours) which have been visualised in Figure 5A. These modules were constructed from a total of 1139 proteins that were found to exhibit significant changes in expression across strains or growth conditions. The Magenta module is the largest, consisting of 244 different proteins, while the Red module is the smallest at just 34 proteins, respectively (Figure 5B). However, most interesting are the Brown and Yellow modules, consisting of 198 and 103 proteins, respectively. These two exhibit particularly strong differential patterns of expression across the strains, particularly when comparing sporulation-inhibited strains to the wildtype, as can be seen in Figure 5C. For further comparisons, summarised expression profiles of all modules are provided in the Supplementary Figure S1. A list of identified proteins for each module has additionally been made available (Table S4).

In the wildtype strain, the proteins captured in the Brown module generally display a strong continuous increase in relative expression across the sampled growth phases, while they consistently appear to be repressed in all the sporulation-inhibited strains. This matches the predicted expression profile of many well-characterised sporulation-related genes. As endospore formation progresses in the wildtype cultures, sporulation-related genes are expected to be increasingly upregulated through the sigma factor cascade. Meanwhile, these same genes would fail to be expressed in sporulation-inhibited strains due to early disruption of the sporulation initiation network. When examining the proteins captured in the Brown module, homologues to regulators σ^E^, σ^F^, and SpoIIID were identified alongside several other sporulation-associated proteins, such as spore coat assembly proteins SpoVM and CotE. This further indicates that the proteins captured by the Brown module are likely to be associated with endospore formation.

In the case of the proteins in the Yellow module, relative expression appears to be consistently upregulated in the wildtype strain and in the Δ*sigF* strain, while the expression seems to be comparatively downregulated in the Δ*spo0A*, Δ*spo0B*, and Δ*spo0F* strains. In the PCA, we observe an apparent divergence between the global proteomic profiles of the Δ*sigF* strain and the other sporulation-inhibited variants, and the Yellow module is a strong reflection of this deviation. Interestingly, the smaller Red module exhibits a similar expression pattern, though less pronounced compared to that of the Yellow module (Figure S1). As was noted in the sigma factor cascade characterisation, the key difference between Δ*sigF* and the other sporulation-inhibited strains is the preserved functionality of the core phosphorelay. The Yellow module encompasses both Spo0A and Spo0F, emphasising its connection to Spo0A and its surrounding regulatory network. As such, this module may include genes involved in the transition into the stationary phase, including sporulation initiation, but could also potentially be linked to other Spo0A-regulated processes, such as cannibalism and biofilm formation.

### 2.5. Module Enrichment and Characterisation

**Figure 6:**
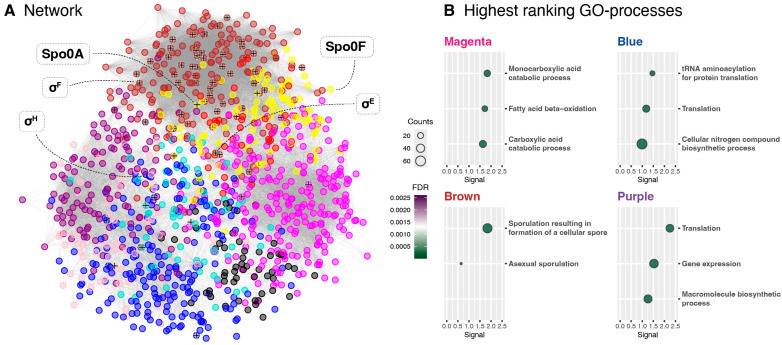
Visualisation of co-expression and enrichments of selected modules from the WGCNA analysis. (**A**) Co-expression network, in which nodes represent specific proteins and edges indicate co-expression (with a threshold of 0.25). Proteins are coloured according to their module assignment. The positions of specific key regulators have been indicated. Furthermore, the *P. thermoglucosidasius* proteins classified under the GO term GO:0030435 “Sporulation resulting in formation of a cellular spore” (as defined by the STRING database [33]) have been labelled with a (+) to emphasise the clustering of sporulation-associated genes in the Brown module. (**B**) The three highest ranking enrichments for the four largest modules, as based on their signal score. “FDR” indicates the false discovery rate, and “Counts” indicates the number of genes identified under each term in the specified module. “Signal” is calculated from the ratio of the observed/expected counts and −log(FDR).

To visualise the relationship between the different WGCNA modules, a co-expression network was generated, and the positions of the core regulators of the sigma factor cascade were identified (Figure 6A). σ^G^ and σ^K^ are not present in the network, as their changes in relative expression were not significant enough to pass the WGCNA filtering. The Green module was excluded from the final network due to its scattered pattern. A complete network with all modules can be viewed in Supplementary Figure S2. Predictably, modules that share similar expression profiles tend to cluster together. This is reflected in the groupings of the Yellow-Red and Turquoise-Blue modules. In contrast, two larger modules, Brown and Magenta, remain more distinct and display stronger internal connectivity. It is also notable that the Brown and Yellow modules localise closely together, supporting the observation that both exhibit expression profiles that are highly correlated.

To gain a better understanding of the functions each module may encompass, gene enrichment was performed through the STRING database [33], and Gene Ontology (GO) terms related to biological processes were extracted for analysis. The Turquoise, Yellow, Red, and Green module enrichments failed to produce any GO-process terms. For the four largest modules, the terms with the three highest signals are displayed in Figure 6B, and a more comprehensive overview of all enriched modules can be found in Supplementary Figure S3. While most of the enriched modules appear to be centred around biosynthetic and metabolic processes, it is apparent that a large part of the Brown module comprises sporulation-associated genes, with approximately 25% of the proteins being directly linked to sporulation-related GO terms. In the STRING database, 167 genes from *P. thermoglucosidasius* are currently coupled to the GO term GO:0030435 (“Sporulation resulting in formation of a cellular spore”). To visualise the accumulation of sporulation genes within the Brown module, all proteins associated with this term were marked in the network in Figure 6A. The result of this enrichment further emphasises that the proteins captured by the Brown module are co-expressed with many sporulation-associated genes and thus are likely to be linked to the sporulation pathway as well.

## 3. Discussion

In this study, we operated under the assumption that the *P. thermoglucosidasius* sporulation pathway is comparable to that of *B. subtilis*. The present investigation of the *P. thermoglucosidasius* sporulation sigma factor cascade provides support for this theory. In our analysis, we found that *P. thermoglucosidasius* carries many homologues of *B. subtilis* sporulation regulators, which exhibit expression patterns largely consistent with those expected from the *B. subtilis* network. Interestingly, homologues of many *B. subtilis* sporulation initiation regulators remain unidentified in the *P. thermoglucosidasius* genome, including several of the kinases that activate the phosphorelay as well as the negative regulators RapABEH. Consequently, little is known about the parameters that govern the entry into the *P. thermoglucosidasius* sporulation pathway, raising the possibility that they may differ from those of *B. subtilis*. As this study did not manage to cover the early sporulation initiation phase in *P. thermoglucosidasius*, more data will need to be collected to unveil the regulators involved in this complex process. In addition, this study confirmed that the deletion of the predicted early-stage regulators Spo0F, Spo0B, Spo0A, and σ^F^ inhibits the sporulation pathway of *P. thermoglucosidasius*, again consistent with what would be expected from *B. subtilis*. Although the gene deletions did not significantly affect the growth rate of the strains, the transition into the stationary phase was more abrupt and occurred at a lower OD than in the wildtype, likely a sign of their disrupted regulation. Notably, microscopy analysis revealed that the deletion of Spo0B did not completely disable sporulation, but still appeared to largely inhibit the process, a pattern for which no equivalent case has been described in *B. subtilis* so far. As only a low amount of sporulation was observed in the Spo0B cultures, the proteomic profiles of the sporulating cells were likely drowned out by the non-sporulating ones during data extraction for this study. More research into the mechanisms that may allow *P. thermoglucosidasius* to bypass the Spo0B regulatory step is therefore necessary.

Through the proteomic analysis, two protein co-expression modules were identified, Brown and Yellow, which displayed expression profiles that were consistent with those expected of sporulation-associated proteins. The presence of the Spo0A and Spo0F key regulators in the Yellow module linked it to early sporulation regulation, while enrichment analysis confirmed the Brown module to encompass many genes related to endospore formation. Though the identification of these modules provides new information about the *P. thermoglucosidasius* sporulation regulatory network, it is also necessary to consider what may not have been sufficiently covered through this proteomic analysis. Notably, protein coverage was not complete, as proteins like Spo0B, σ^G^, and σ^K^ were lost during protein extraction or data filtering. In addition, proteomics analysis is generally not sensitive enough to recover everything, including smaller peptides such as toxins. Due to these inherent limitations, future studies could employ complementary transcriptomics or targeted approaches (i.e., parallel reaction monitoring or qPCR) to validate and further investigate the regulatory modules identified in this study. For many proteomic characterisations, it is also preferable to inoculate each replicate from separate cultures to account for intrastrain variance. However, to facilitate synchronisation between the different growth phases for each strain, we elected to inoculate all replicates from the same pre-culture, since this results in more consistent growth patterns between replicates. As such, the results of this study likely do not encompass the entire regulatory profile of the sporulation pathway. To fully comprehend the sporulation network of *P. thermoglucosidasius*, more data will therefore need to be generated.

## 4. Materials and Methods

### 4.1. Strains, Plasmids, and Media

Plasmids and strains used in this study have been listed in Supplementary Table S1 and Table S2, respectively.

Escherichia coli DH5α was used as a host for cloning plasmids. *E. coli* strains were routinely plated on LB agar plates and cultured in Lysogeny Broth (LB) medium at 37 °C under continuous agitation. *P. thermoglucosidasius* DSM2542 strains were generally cultured in Salt Peptone Yeast (SPY) medium at 60 °C under agitation. The SPY medium contained, per litre: 16 g soy peptone, 10 g yeast extract, and 5 g NaCl, with the final pH of the medium adjusted to 6.8. Furthermore, *P. thermoglucosidasius* strains were grown on Trypticase Soy Agar (TSA) plates (BD Biosciences, Franklin Lakes, NJ, USA). For the selection of transformants, *E. coli* DH5α and *P. thermoglucosidasius* DSM2542 were grown with kanamycin (Km) at concentrations of 6.25 mg/L and 12.5 mg/L, respectively.

For growth and induction of the sporulation pathway, *P. thermoglucosidasius* strains were cultured in Thermophile Minimal Medium (TMM) supplemented with 1 g/L yeast extract. TMM was prepared as described by Fong et al. [34], with some modifications. Each litre of medium consisted of 930 mL Six Salts Solution (SSS), 40 mL of 1 M MOPS solution (pH adjusted to 8.2), 10 mL of 1 mM FeSO_4_ in 0.4 M tricine, 10 mL of 0.132 M K_2_HPO_4_, 10 mL of 0.953 M NH_4_Cl, 0.5 mL of 1 M CaCl_2_, 0.5 mL of trace elements solution, and 1 mL of Wolfe’s vitamin solution. The SSS were prepared as follows: 4.95 g NaCl, 1.45 g Na_2_SO_4_, 0.25 g KCl, 0.04 g KBr, 1.85 g MgCl_2_·6H_2_O, and 0.89 g NaNO_3_, per litre. The trace-element solution was composed by 1 g FeCl_3_·6H_2_O, 0.18 g ZnSO4·7H2O, 0.12 g CuCl_2_·2H_2_O, 0.12 g MnSO_4_·H2O, and 0.18 g CoCl_2_·6H_2_O, per litre. The Wolfe’s vitamin solution contained 10 mg Pyridoxine HCl, 5 mg Thiamine HCl, 5 mg Riboflavin, 5 mg Nicotinic acid, 5 mg Ca-D-(+)pantothenate, 5 mg p-Aminobenzoic acid, 5 mg Thiotic acid (Dithiolane Pentanoic acid), 2 mg Biotin, 2 mg Folic acid, and 0.1 mg Vitamin B12, per litre. The medium pH was adjusted to 6.8.

### 4.2. Annotation Collection

To investigate the protein functionalities of *P. thermoglucosidasius* DSM2542, genomic annotation data was collected from NCBI-managed databases GenBank [35] and RefSeq [36], under accession numbers CP012712.1 and NZ_CP012712.1, respectively. In addition, functional orthologous matches to the GenBank genome reference were extracted from the KEGG database [37]. Based on the collected annotations, the target genes *spo0A*, *spo0B*, *spo0F*, and *sigF* were identified under the GenBank locus tags AOT13_16410, AOT13_17465, AOT13_03630, and AOT13_16100, respectively. Proteome sequences were collected from the UniProt archive (UniParc) [38] under proteome ID UP000033360, and were then linked to GenBank locus tags based on matching sequences.

### 4.3. Plasmid Construction

Three plasmids were designed for the purpose of deleting sporulation genes *sigF*, *spo0B*, and *spo0F* in *P. thermoglucosidasius* DSM2542. Fragments for each knockout plasmid were amplified using the primers listed in Supplementary Table S3, and homologous arms were amplified from *P. thermoglucosidasius* DSM2542 gDNA.

USER cloning (New England Biolabs, Ipswich, MA, USA) was used for plasmid assembly. Each reaction was prepared by mixing the required plasmid fragments with 1.2 µL of 10× CutSmart Buffer (New England Biolabs, Ipswich, MA, USA) and DNase/RNase-free water, bringing the volume to 11 µL. The reaction was initiated by adding 1 µL of USER enzyme (New England Biolabs, Ipswich, MA, USA), and was then first incubated at 37 °C for 25 min and then at 25 °C for 25 min. 8 µL of DNase/RNase-free water was subsequently added to each reaction, and 5 µL of these final mixtures were then transformed into chemically competent *E. coli* DH5α-λpir (Table S2). The transformed cells were plated on LB-Km and left to incubate overnight at 37 °C, except for pMM5 transformants, which were incubated at 30 °C. Single colonies containing correctly assembled plasmids were identified through colony PCR with primer set 11M2/12 (Table S3), using OneTaq Quick-Load 2X Master Mix with Standard Buffer (New England Biolabs, Ipswich, MA, USA) following manufacturers’ instructions. The plasmids were subsequently purified from the selected colonies using a NucleoSpin Plasmid kit (Macherey-Nagel, Düren, Germany) and verified through sequencing (Eurofins Genomics, Ebersberg, Germany) using primers 11M2 and 12.

### 4.4. Transformation of P. thermoglucosidasius

*P. thermoglucosidasius* DSM2542 cells were made competent by inoculating loopfuls of the strain into a shake flask containing 50 mL of SPY pre-heated to 60 °C. The cell culture was incubated at 60 °C and 200 rpm until it reached an OD600 of approximately 1.5. Following this, the cells were reinoculated to an OD600 of 0.5 in a new shake flask containing 30 mL of fresh pre-heated SPY medium. The cells were again incubated at 60 °C and 200 rpm until reaching an OD600 of approximately 1.7. The resulting culture was subsequently incubated on ice for 10 min, after which the culture was split into two tubes. The two culture tubes were both spun down at 4000× *g* in a 4 °C centrifuge, and the pellets were resuspended in 15 mL of ice-cold electroporation buffer (0.5 M mannitol, 0.5 M sorbitol, 10% glycerol). Both cultures were then washed through three rounds of cooled centrifugation and resuspension in 10, 10, and 5 mL of ice-cold electroporation buffer, respectively. Following the final round of centrifugation, both cultures were gently resuspended in 2 mL of electroporation buffer. These final competent cell suspensions were then transferred as 60 µL aliquots to tubes pre-chilled on dry ice, after which they were frozen and stored at −80 °C.

For transformations, competent cell aliquots were removed from −80 °C storage and allowed to thaw on ice. Next, 2.5 µL of plasmid was transferred to each cell aliquot. The cell-plasmid mixtures were then transferred to electroporation cuvettes pre-chilled on ice. The cuvettes were shocked with an exponential pulse with a voltage of 2500 V, capacitance of 10 µF, and resistance of 600 Ω. Immediately following the shock, the cells were transferred to 1 mL of SPY supplied with 1% glycerol and pre-heated to 52 °C. The transformations were subsequently incubated for 3 h at 52 °C and 250 rpm. Following this, the cells were spun down at 3000 g and plated on TSA-Km. The plates were then left to incubate overnight at 52 °C.

### 4.5. Gene Deletion in P. thermoglucosidasius

To delete the target genes, each knockout plasmid (Table S1, Figure S4) was first transformed into the *P. thermoglucosidasius* wildtype strain. Plasmid integration was achieved by inoculating the successfully transformed colonies in 2 mL of SPY with 12.5 mg/L kanamycin. The cultures were incubated overnight at 62 °C with agitation at 250 rpm. Subsequently, cells were streaked onto TSA-Km and incubated overnight at 60 °C.

Colony PCR was used to verify the successful integration of the plasmids. Colony PCR was performed by resuspending part of the single colonies in 20 mM NaOH and heating the suspensions to 95 °C for 10 min. After being cooled to room temperature, 1 µL of suspension was used as the template for the colony PCRs. Plasmid integration was checked by colony PCR using OneTaq Quick-Load 2X Master Mix with Standard Buffer (New England Biolabs, Ipswich, MA, USA), as per the manufacturer’s protocol.

Once the successfully integrated colonies had been identified, gene deletion was performed by inoculating each colony into 2 mL of SPY and incubating it overnight at 60 °C, 250 rpm. To facilitate loop out of the knockout plasmids, the resulting cultures were repeatedly passaged to fresh medium over the course of two days. The passaging was performed by transferring 500 µL of each growing culture into 1.5 mL of fresh pre-heated SPY during the early morning and late afternoon. During the afternoon of the second day, all cultures were diluted and plated onto TSA plates, which were then left to incubate overnight at 60 °C. The resulting plates were then observed under blue light to detect successful recombinants through their lack of fluorescence. For the *sigF* gene deletion, successful recombinants were identified via replica plating on TSA and TSA-Km. Recombinant colonies were isolated and screened for deletion of the target gene through colony PCR. Colonies displaying the predicted band sizes were then finally confirmed as successful knockouts via sequencing.

### 4.6. Microscopy

1% agarose was used to prepare agar pads on the glass slides for the purpose of immobilising the cells. For microscopy, 5 µL of culture was placed on an agar pad and topped with a glass coverslip. Phase contrast microscopy was performed using a Leica DM4000 B microscope (Leica Microsystems, Wetzlar, Germany) equipped with a 63× oil immersion objective. Image acquisition was performed using a Leica DFC300 FX camera (Leica Microsystems, Wetzlar, Germany) operated through the Leica Application Suite software (v4.12.0).

### 4.7. Growth and Proteomics Sampling

To investigate the impact of the sporulation-associated deletions on the metabolism of *P. thermoglucosidasius*, we examined the proteome of the knockout strains during various stages of growth. To maintain synchronisation during the different growth and sporulation phases, strain replicates were inoculated from the same pre-cultures, yielding one biological replicate per strain. Pre-cultures for each strain were inoculated with a loopful of cells collected from the TSA plates. The wildtype, Δ*spo0A*, Δ*spo0B*, Δ*spo0F*, and Δ*sigF* strains were then each pre-cultured at 60 °C and 200 rpm in shake flasks containing 30 mL of SPY. The following day, each pre-culture was transferred to an OD600 of 0.1 in three separate shake flasks containing 50 mL TMM supplied with 1 g/L yeast extract, providing three technical replicates per strain.

Following inoculation into TMM, the cultures were grown for 24 h at 60 °C, 200 rpm. During this period, OD600 measurements were performed hourly to monitor growth. Meanwhile, proteomic samples were collected once the cultures reached the exponential, transition, and stationary growth phases (see Table S7). For sample collection, 1 mL of culture was spun down at 4000 g for 7 min, after which the supernatant was separated from the pellet. The pellet was subsequently stored at −21 °C.

### 4.8. HPLC-MS and Data Processing

Samples collected for proteomics analysis were thawed on ice, and proteins were lysed using a lysis buffer containing 6 M guanidinium hydrochloride, 5 mM tris(2-carboxyethyl)phosphine (TCEP), 10 mM 2-chloroacetamide (CAA), and 100 mM Tris·HCl, pH 8.5, with the addition of two 3 mm zirconium oxide beads. The samples were disrupted using a Mixer Mill (Retsch GmbH, Haan, Germany) set to 25 Hz for 5 min at ~20 °C, followed by heating for 10 min at 99 °C in a ThermoMixer (Eppendorf, Hamburg, Germany) (1800 rpm). Cell debris was removed by centrifugation at 15,000× *g* for 10 min at 23 °C, and a 50 µL aliquot of the supernatant was collected and diluted with 50 µL of 50 mM ammonium bicarbonate.

Protein concentration was measured using the bicinchoninic acid (BCA) assay. For tryptic digestion, 20 µg of protein was diluted to a final volume of 100 µL with 50 mM ammonium bicarbonate. Trypsin and LysC were added at a final concentration of 0.1 µg/µL (1:1 ratio), and digestion was carried out at 37 °C for 16 h with constant shaking at 400 rpm. The digestion was terminated by adding 10 µL of 10% trifluoroacetic acid (TFA), followed by centrifugation at 15,000× *g* for 15 min at 23 °C. Peptides were desalted using StageTips packed with C18 resin and were dried by vacuum centrifugation. The peptides were reconstituted in 0.1% formic acid prior to high-performance liquid chromatography-mass spectrometry (HPLC-MS) analysis.

HPLC-MS was carried out on a Dionex UltiMate 3000 system (Thermo Fisher Scientific, Waltham, MA, USA) coupled to an Orbitrap Exploris 480 mass spectrometer (Thermo Fisher Scientific, Waltham, MA, USA) operated in data-dependent acquisition mode. Samples were first loaded onto a μ-precolumn (C18 PepMap 100, 5 µm, 100 Å) at a 10 µL/min flow rate. Peptides were then resolved on a 15 cm C18 analytical column (PepMap RSLC C18, 2 µm, 100 Å, 150 µm × 15 cm) using a 60 min gradient from 4 to 76% (*v*/*v*) acetonitrile in water containing 0.1% formic acid, at a 1.2 µL/min flow rate.

Raw data from HPLC-MS were processed using Proteome Discoverer v2.4 (Thermo Fisher Scientific, Waltham, MA, USA). The following parameters were used for data analysis: precursor mass tolerance was set to 10 ppm, fragment mass tolerance to 0.02 Da, and trypsin (full) was selected as the digestion enzyme, allowing for up to two missed cleavages. Peptides with lengths between 6 and 144 amino acids were considered, and the false-discovery rate (FDR) was controlled at 0.1%. For protein identification, sequences were searched against the UniProt database (The UniProt Consortium, 2021) [38].

### 4.9. Differential Expression Analysis

The resulting quantification data were further processed using the DEP package (version 1.20.0) [39] in RStudio (version 2024.04.2) for differential expression analysis. First, proteins were filtered to only include proteins that were quantified in all replicates of at least one condition. Normalisation was performed using variance stabilisation normalisation (VSN) to account for technical variability, followed by imputation of missing values. Statistical testing for differential protein expression was assessed with a false discovery rate (FDR) threshold of 0.05 and log_2_ fold changes (LFC) threshold of 1.5. Heatmaps were generated using the sechm package (version 1.6.0) in RStudio.

### 4.10. Weighted Gene Co-Expression Network Analysis (WGCNA) and Enrichment

Based on the data derived from the differential expression analysis, average values over three technical replicates were used for gene co-expression network analysis utilising R package WGCNA (version 1.72-5) [40]. The network topology was assessed and the soft-threshold power of 10 was selected to achieve a scale-free topology fit (R^2^ > 0.8). Modules were then detected using the Topological Overlap Matrix and hierarchical clustering with a minimum size of 30 proteins and a merge cut-height of 0.25. Module–trait relationships were calculated using module eigengenes and Pearson correlations.

Network visualisation was performed with Cytoscape (version 3.10.2) [41] with edge weight filtered to 0.25 or above. Module enrichment was accomplished through the STRING database [33]. The analysis was run with an FDR stringency of 5% on the full STRING network, specifying *P. thermoglucosidasius* as the reference organism. Data regarding Gene Ontologies (GOs) for biological processes were subsequently extracted for each module.

## Data Availability

The mass spectrometry proteomics data have been deposited to the ProteomeXchange Consortium via the PRIDE [42] partner repository with the dataset identifier PXD069977 and 10.6019/PXD069977. During review use 2FYuA7SaiO7c token to access the data.

## Supplementary Materials

The following supporting information can be downloaded at: https://www.mdpi.com/article/10.3390/ijms262311735/s1.

## Abbreviations

The following abbreviations are used in this manuscript:

PCA	Principal Component Analysis
SPY	Salt Peptone Yeast medium
SSS	Six Salts Solution
TMM	Thermophile Minimal Medium
TSA	Trypticase Soy Agar
WGCNA	Weighted Gene Co-Expression Network Analysis
HPLC-MS	High Performance Liquic Chromatography-Mass spectometry
