# Supplementary material for: Analysis of Proteome Dynamics in Early-Stage Sporulation-Inhibited Variants of Parageobacillus thermoglucosidasius
Source: Int J Mol Sci. 2025 Dec 4;26(23):11735. doi: 10.3390/ijms262311735 (PMC12692396; doi:10.3390/ijms262311735)
Supplement: Supplementary file 1 [file ijms-26-11735-s001.zip › Millgaard_et_al_Supplementary_Material.pdf]

Analysis of proteome dynamics in early-stage sporulation-inhibited variants of *Parageobacillus thermoglucosidasius*

Marie Millgaard[1], Oihane Irazoki[1], Viviënne Mol[1], Ivan Pogrebnyakov[1], Alex Toftgaard Nielsen[1]

[1] *Technical University of Denmark, The Novo Nordisk Foundation – Center for Biosustainability, Kgs. Lyngby, Denmark.*

Supplementary Material

- **Table S1:** Plasmids used in this study
- **Table S2:** Strains used in this study
- **Table S3:** Primers used in this study
- **Table S4:** Summary of proteins associated to each WGCNA module, including tags, annotations, and descriptions obtained from UniParc, GenBank (GB), RefSeq (RS), and KEGG.
- **Table S5:** All STRING enrichments obtained from each WGCNA module.
- **Table S6:** P. thermoglucosidasius DSM2542 proteome annotation file used in this study, as based on information collected from UniParc, GenBank (GB), RefSeq (RS), and KEGG.
- **Table S7:** Metadata for all proteomic samples analysed in this study.
- **Figure S1:** Expression profiles of all modules identified during the WGCNA
- **Figure S2:** Full co-expression network depicting the modules identified through WGCNA.
- **Figure S3:** The twenty highest ranking GO-process terms for all enriched modules, as based on signal scores
- **Figure S4:** Maps of knockout plasmids for *spo0B*, *spo0F*, *spo0A*, and *sigF*.

Table S4: *Plasmids used in this study. "KO" indicates that the plasmid is carrying flanking regions of the specified gene for the purpose of deleting it.*

Plasmid	Description	Source
pGB-sfGFP-best	repB-sfGFP-KanR-ColE1-$ptsI_{KO}$	Millgaard et al.[1]
pVM55	repB-KanR-ColE1-$sigF_{KO}$	This work
pMM5	repB-sfGFP-KanR-ColE1-$spo0B_{KO}$	This work
pMM6	repB-sfGFP-KanR-ColE1-$spo0F_{KO}$	This work
pMM7	repB-sfGFP-KanR-ColE1-$spo0A_{KO}$	Millgaard et al.[1]

Table S5: *Strains used in this study.*

Strain	Relevant genotype or properties	Source
E. coli DH5α-λ*pir*	F- ϕ80*lac*ZΔM15 Δ(*lac*ZYA-*arg*F)U169 *rec*A1 *end*A1 *hsd*R17(rk-, mk+) *pho*A *sup*E44 *thi*-1 *gyr*A96 *rel*A1 λ-*pir* lysogen of DH5α	Lab collection
P. thermoglucosidasius DSM2542	Wildtype isolate	Bacillus Genetic Stock Center (USA)
Pth::pVM55	DSM2542::pVM55 Kan^R	This work
Pth::pMM5	DSM2542::pMM5 Kan^R	This work
Pth::pMM6	DSM2542::pMM6 Kan^R	This work
Pth::pMM7	DSM2542::pMM7 Kan^R	This work
Pth Δ*sigF*	DSM2542 Δ*sigF*	This work
Pth Δ*spo0B*	DSM2542 Δ*spo0B*	This work
Pth Δ*spo0F*	DSM2542 Δ*spo0F*	This work
Pth Δ*spo0A*	DSM2542 Δ*spo0A*	Millgaard et al.[1]

[1] Millgaard M, Bidart GN, Pogrebnyakov I, Nielsen AT, Welner DH. An improved integrative GFP-based vector for genetic engineering of *Parageobacillus thermoglucosidasius* facilitates the identification of a key sporulation regulator. AMB Express 2023;13. https://doi.org/10.1186/s13568-023-01544-9.

Table S6: *Primers used in this study. Overhangs designed for assembly during USER cloning are underlined.*

No.	Sequence 5'-3'	Description
23	ACCCGGGGUTCCTCTAG	Forward primer to amplify plasmid backbone
24d	AATTCGUAATCATGGTCATATGGATACAGCG	Reverse primer to amplify plasmid backbone
MM1	ACGAATUCATTAAAACAATAATGGTGGTTTCACGAAAAAGGACTATC	Forward primer to amplify Δ*spo0F* left homologous arm
MM2	ACACACCUCAATTCTTTCATTTATTTTGTTCCATGTTATAAG	Reverse primer to amplify Δ*spo0F* left homologous arm
MM3	AGGTGTGUCTATAAGGAAAGTTGTCACGTTTTCCTTGAGTTACGAGG	Forward primer to amplify Δ*spo0F* right homologous arm
MM4	TCCCCGGGUCCATTTCCGCAAATCCCAGTTTTGGTTC	Reverse primer to amplify Δ*spo0F* right homologous arm
MM5	ACGAATUTCGCGCGTTTACGATGAAAGGG	Forward primer to amplify Δ*spo0B* left homologous arm
MM6	ACAGCGCUCCCATTCATAAATTACACCG	Reverse primer to amplify Δ*spo0B* left homologous arm
MM7	AGCGCTGUGGCATGGAGGCAGAGCTGTTTTAGGAATCA	Forward primer to amplify Δ*spo0B* right homologous arm
MM8	ATCCCCGGGUTGGGACAAGCGTCGTAAAATGATATTCGG	Reverse primer to amplify Δ*spo0B* right homologous arm
P153	ACGAATUCATCCGGCTTTCCGGAGA	Forward primer to amplify Δ*sigF* left homologous arm
P154	ATGCTUAGTTACATAACGCTTTTTGGCT	Reverse primer to amplify Δ*sigF* left homologous arm
P155	AAGCAUCCATTCTTTTTTATTTTTATGAAAT	Forward primer to amplify Δ*sigF* right homologous arm
P156	ATCCCCGGGUTACTTTCTTTGTTTTCATATAAAAT	Reverse primer to amplify Δ*sigF* right homologous arm
11M2	GGCCGCTGTATCCATATGACCATG	Forward primer for sequencing homologous arms region in plasmids
12	GTTGTAAAACGACGGCCAGTGC	Reverse primer for sequencing homologous arms region in plasmids
MM11	TTGTCGCTTCCCAGTTCCATCCG	Forward primer for sequencing of *spo0F* genomic region
MM12	GGCGCGCTGGATTTGTTCTGTCG	Reverse primer for sequencing of *spo0F* genomic region
MM13	CAAACAAGGTCACCGTCAGCCTTACAC	Forward primer for sequencing of *spo0B* genomic region
MM14	CTTGATGCGCGCCTTCAATCAGC	Reverse primer for sequencing of *spo0B* genomic region
MM15	ACGCCACAAATCCTGTTCCG	Forward primer for sequencing of *sigF* genomic region
MM16	CCCGCATGTTGTAGCGAAACTTC	Reverse primer for sequencing of *sigF* genomic region

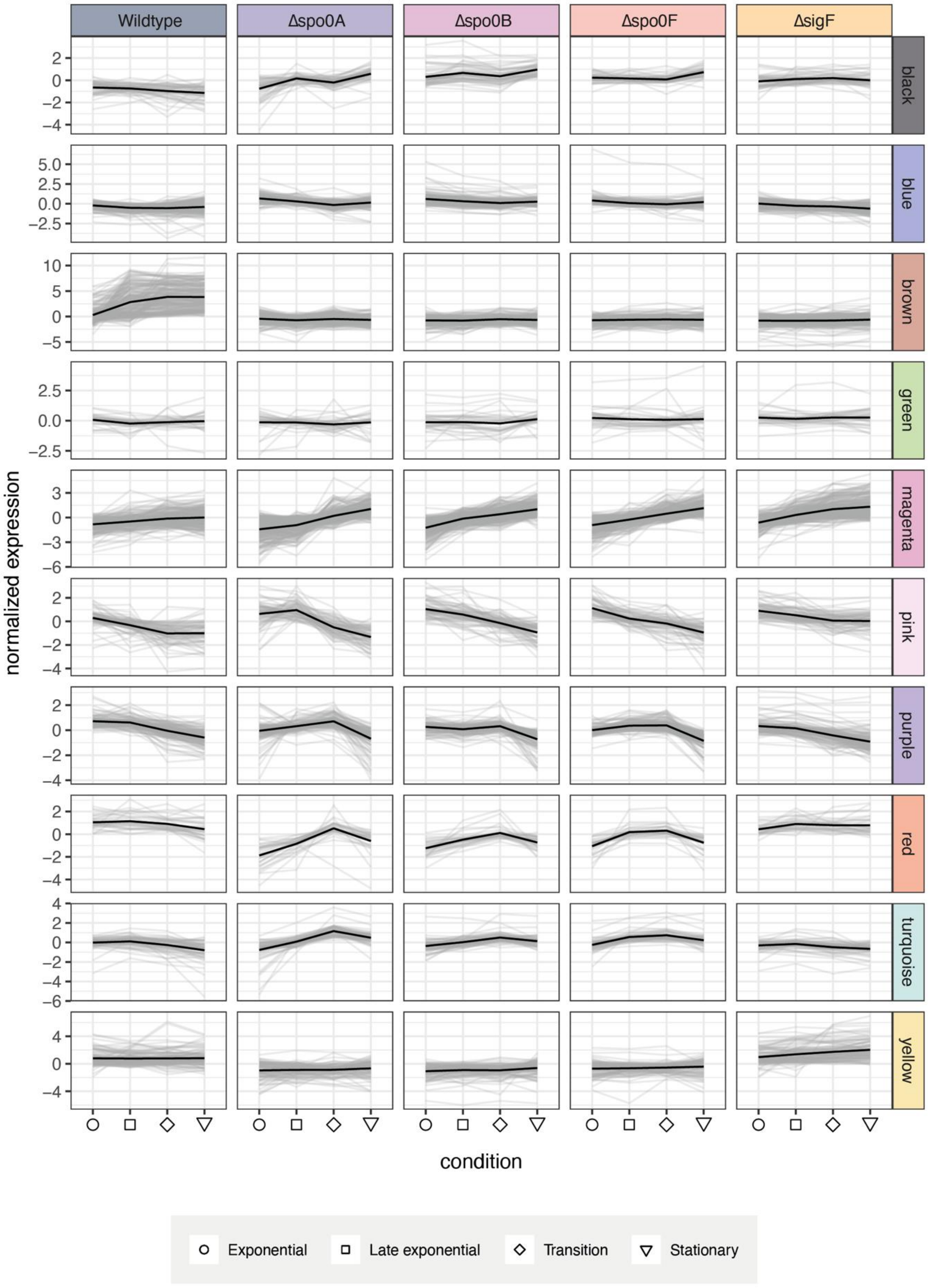

***Figure S1:** Expression profiles of all modules identified during the WGCNA.*

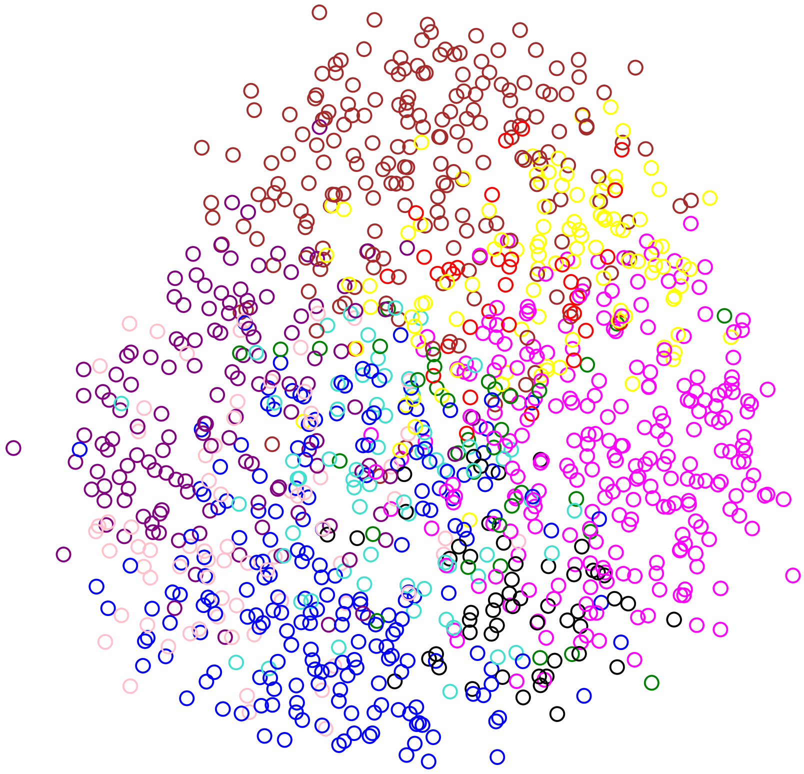

Figure S2: *Full co-expression network depicting the modules identified through WGCNA.*

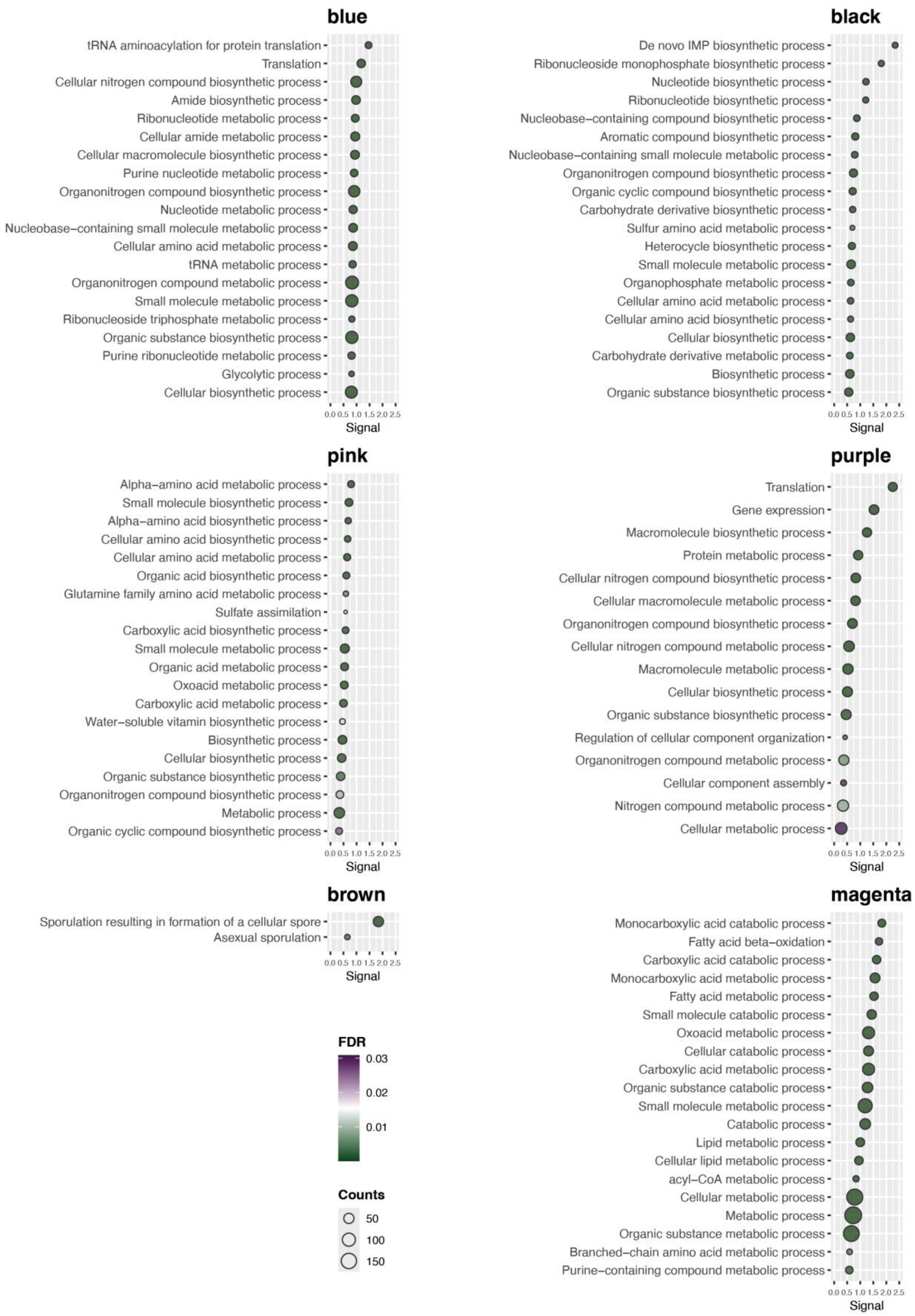

Figure S3: *The twenty highest ranking GO-process terms for all enriched modules, as based on signal scores. "FDR" indicates the false discovery rate, and "Counts" indicate the number of genes identified under each term in the specified module. "Signal" is calculated from the ratio of the observed/expected counts and -log(FDR).*

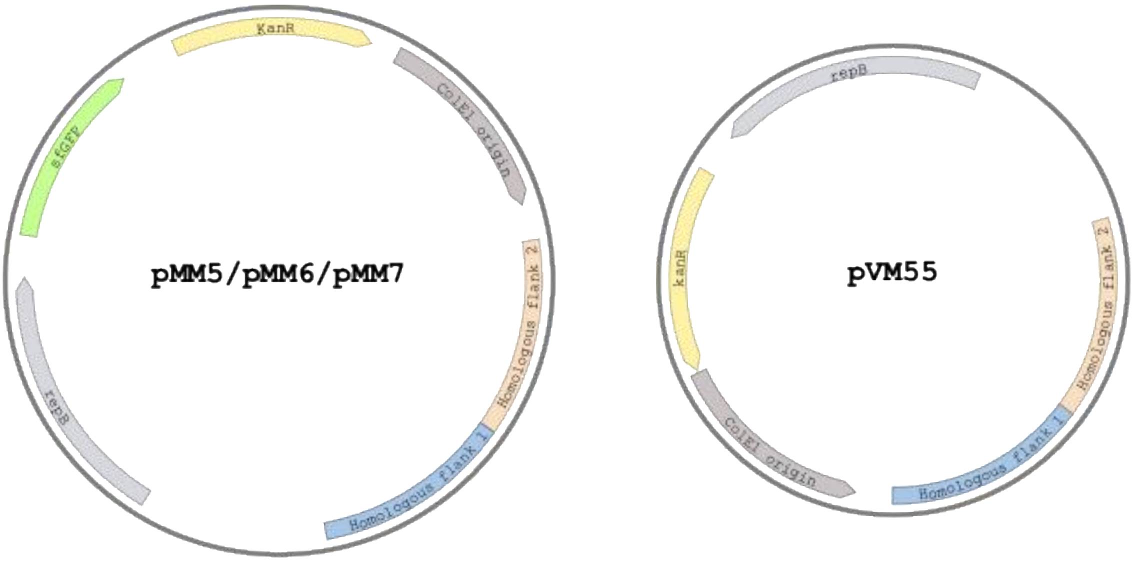

Figure S4: *Maps of knockout plasmids for spo0B (pMM5), spo0F (pMM6), spo0A (pMM7), and sigF (pVM55). The flank sequences are homologous to the left and right flanks of the targeted deletion sites.*
